# Vaginal Myeloid Sarcoma: A Rare Extramedullary Presentation of a Myeloid Neoplasm

**DOI:** 10.7759/cureus.94466

**Published:** 2025-10-13

**Authors:** Arif Onur Atay, Ece Sen, Ajda Gunes, Mine Hekimgil, Ali Akdemir

**Affiliations:** 1 Obstetrics and Gynecology, Torbali State Hospital, Izmir, TUR; 2 Obstetrics and Gynecology, Ege University Faculty of Medicine, Izmir, TUR; 3 Hematology, Ege University Faculty of Medicine, Izmir, TUR; 4 Pathology, Ege University Faculty of Medicine, Izmir, TUR

**Keywords:** acute myeloid leukemia, extramedullary myeloid tumor, gynecologic neoplasm, rare case report, vaginal myeloid sarcoma

## Abstract

Myeloid sarcoma is a rare extramedullary tumor often associated with acute myeloid leukemia (AML), but it can also appear as an isolated lesion. Vaginal involvement is extremely rare and may lead to delayed diagnosis due to nonspecific symptoms. We report a 41-year-old woman with a history of two cesarean sections who presented with dyspareunia. A firm, fixed mass was palpated on the posterior vaginal wall. Transvaginal ultrasonography and pelvic MRI revealed a 7.5 × 4.3 cm mass in the rectovaginal space without rectal infiltration. The lesion was excised vaginally, and intraoperative endoscopy confirmed rectal integrity. Histopathology showed blast cell infiltration with strong CD7, CD38, CD43, CD45, and BCL-2 positivity, consistent with myeloid sarcoma. Bone marrow biopsy confirmed AML. The patient was treated with 16 cycles of chemotherapy and underwent allogeneic bone marrow transplantation. This case illustrates the diagnostic challenge of vaginal myeloid sarcoma and emphasizes the role of imaging, immunohistochemistry, and multidisciplinary management in such rare presentations.

## Introduction

Vaginal myeloid sarcoma is an exceptionally rare and aggressive form of neoplasm characterized by the aberrant proliferation of immature myeloid precursors in extramedullary sites, particularly within the female genital tract. This malignancy is primarily associated with acute myeloid leukemia (AML), occurring either concurrently with it or as a solitary manifestation in patients with no prior hematological malignancy, making its diagnosis challenging and often delayed [1]. The term "myeloid sarcoma" encompasses various presentations of this tumor, which can appear in discrete anatomical locations, including the vagina, where it is recognized as one of the rarest sites of involvement [2,3]. Fewer than 20 cases of primary vaginal myeloid sarcoma have been reported in the literature to date; extramedullary involvement occurs in approximately 2-9% of acute myeloid leukemia cases [2,4]. The clinical symptoms of vaginal myeloid sarcoma typically include vaginal bleeding, discharge, and, in some cases, the presence of a palpable mass [4]. Histologically, vaginal myeloid sarcoma exhibits a spectrum of growth patterns, distinguishing itself from other sarcomas through characteristic findings, including the presence of myeloblasts and specific immunohistochemical markers such as positivity for myeloperoxidase. Accurate pathological assessment is essential, as incorrect diagnosis may lead to inadequate treatment regimens. Reports indicate myeloid sarcoma can present with extensive local invasion and marked cellular atypia, making its recognition vital for prompt therapeutic intervention [5,6]. Recent literature details several case reports highlighting the significant complexities in diagnosing vaginal myeloid sarcoma, often requiring a multidisciplinary approach for both diagnosis and management. Furthermore, management strategies often involve a combined approach of surgical excision and adjuvant chemotherapy to address systemic disease [7]. In summary, the identification of vaginal myeloid sarcoma remains a considerable diagnostic challenge due to its rarity and symptomatic overlap with more common gynecological disorders. Ongoing research and case documentation are critical to enhance awareness and understanding of this complex malignancy, as well as to refine the therapeutic landscape for affected patients. Timely recognition of vaginal myeloid sarcoma can guide early diagnosis of underlying hematologic malignancy and potentially improve prognosis.

## Case presentation

**Figure 1:**
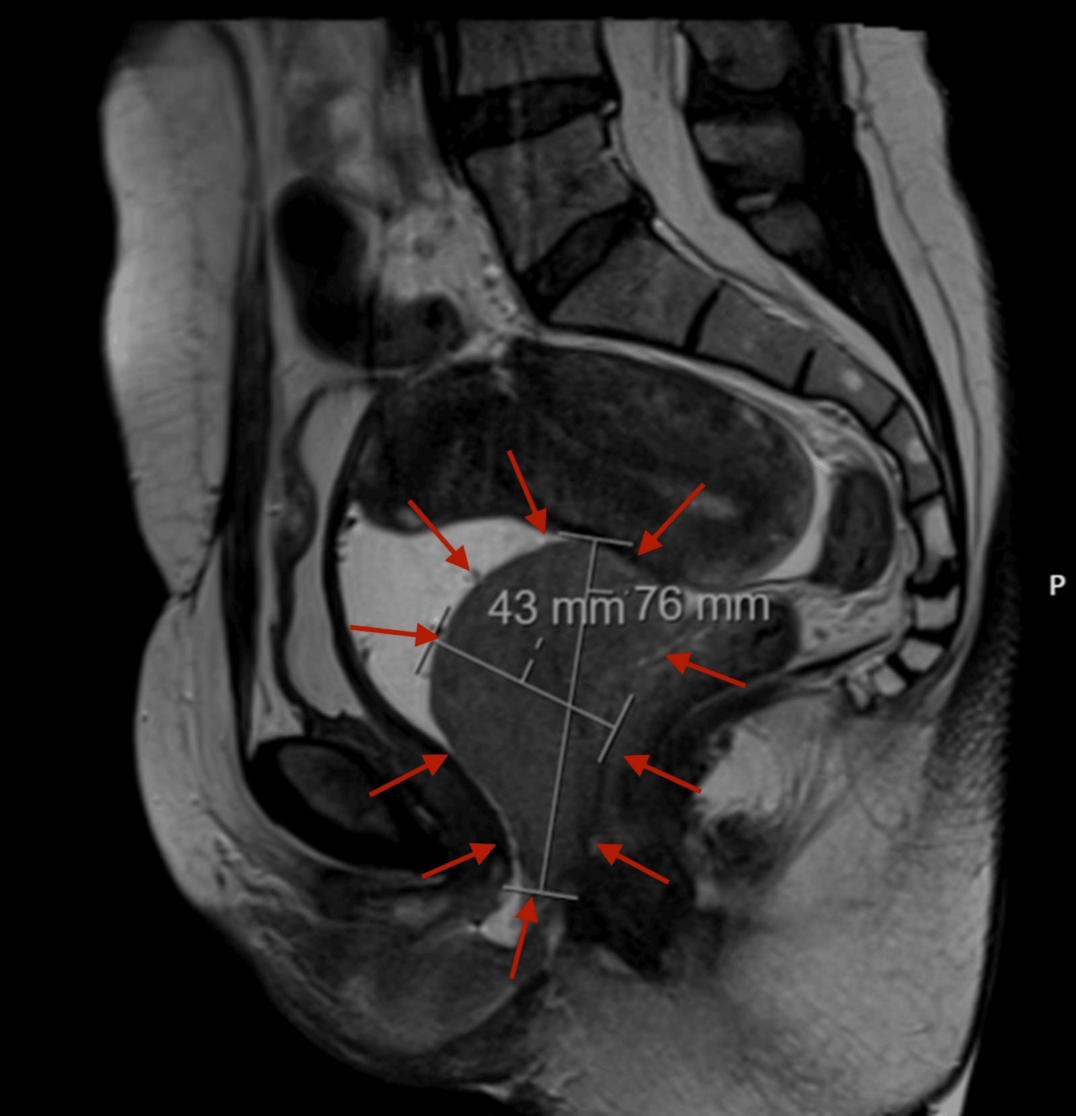
Sagittal pelvic MRI showing a solid mass occupying the rectovaginal space Sagittal T2-weighted pelvic magnetic resonance imaging (MRI) demonstrating a well-defined solid mass measuring 76 × 43 mm (indicated by red arrows) arising from the posterior vaginal wall and extending superiorly toward the cervix. The lesion caused anterior compression of the bladder and posterior displacement of the rectum. During the MRI examination, the vaginal cavity was filled with ultrasound gel to better delineate the lumen, which is clearly visible anterior to the mass. This finding confirms that the lesion originates posterior to the vaginal lumen, consistent with an extramedullary myeloid sarcoma.

**Figure 2:**
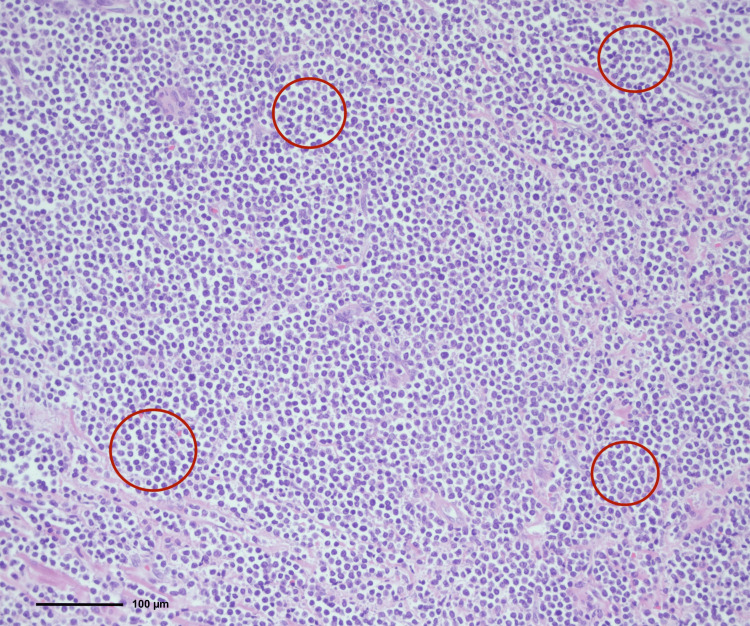
Histopathological examination of the vaginal mass showing diffuse submucosal infiltration of blast cells (H&E, ×20) Hematoxylin and eosin (H&E)-stained section of the vaginal mass showing a diffuse and dense infiltration of immature myeloid blast cells throughout the submucosal connective tissue (highlighted by open circles) (original magnification ×20; scale bar: 100 µm). The tumor cells are monomorphic, medium-sized, and arranged in a sheet-like growth pattern beneath the surface epithelium, characterized by a high nuclear-to-cytoplasmic ratio, fine chromatin, scant cytoplasm, and inconspicuous nucleoli. This diffuse infiltration pattern leads to partial effacement of the normal stromal architecture, a histopathological hallmark of extramedullary myeloid sarcoma. The surface squamous epithelium remains intact, and the neoplastic infiltrate is confined to the submucosa, supporting the tumor’s origin in the posterior vaginal wall.

**Figure 3:**
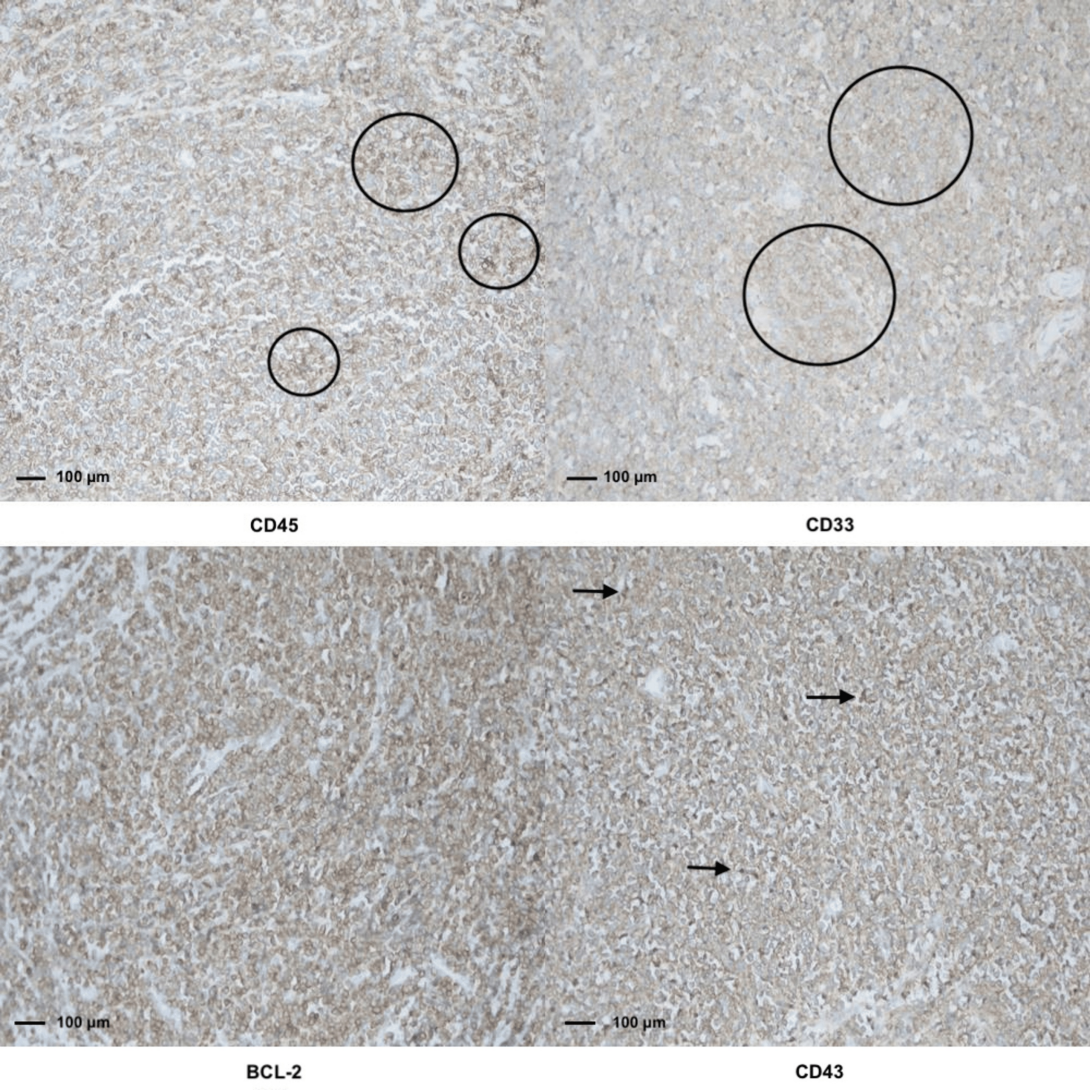
Immunohistochemical staining of the vaginal mass showing strong positivity for CD45, CD33, BCL-2, and CD43 (×20) Immunohistochemical analysis of the vaginal mass demonstrating the myeloid differentiation of tumor cells (original magnification ×20; scale bar: 100 µm for each panel). Each panel represents staining for a specific immunomarker. Black circles indicate areas of positive immunostaining for CD45 (upper left) and CD33 (upper right), highlighting the hematopoietic origin of the neoplastic cells. A black arrow identifies a representative area of CD43 expression (lower right), further supporting myeloid lineage. Diffuse cytoplasmic BCL-2 expression is evident throughout the tumor tissue in the lower left panel, demonstrating increased anti-apoptotic activity, and is therefore not specifically annotated. Together, these findings confirm the myeloid nature of the neoplastic infiltration, complementing the histomorphological features and supporting the diagnosis of extramedullary myeloid sarcoma.

A 41-year-old woman with a history of two prior cesarean deliveries and no comorbid conditions presented with progressive pelvic pain and dyspareunia lasting for approximately three months. Physical examination revealed a firm, fixed mass in the vaginal wall. Baseline hematologic tests revealed normal results, including hemoglobin of 13.7 g/dL, white blood cell count of 10.08 × 10⁹/L, and platelet count of 325 × 10⁹/L. A bone marrow biopsy performed subsequently confirmed acute myeloid leukemia (AML) with myeloblastic differentiation. Transvaginal ultrasonography demonstrated a 7 × 5 cm solid lesion located in the vaginal wall. Pelvic magnetic resonance imaging confirmed a 7.5 × 4.3 cm solid mass occupying the rectovaginal space without evidence of rectal infiltration (Figure 1). A consultation with general surgery was requested, and rigid rectosigmoidoscopy was performed, which showed no mucosal invasion. Surgical excision of the mass was planned and performed via a vaginal approach. A posterior vaginal incision was made, and careful dissection allowed complete excision of the lesion. Intraoperative rectosigmoidoscopy confirmed the integrity of the rectum. The patient was discharged on postoperative day 2 without complications. Histopathological evaluation revealed diffuse infiltration of blast cells in the submucosa (Figure 2) with strong positivity for CD7, CD38, CD43, CD45, and BCL-2, mild positivity for CD33, and partial positivity for CD79a, CD68, CD117, and PAX5, consistent with myeloid sarcoma (Figure 3). Postoperative hematological assessment showed no hepatosplenomegaly or lymphadenopathy. Bone marrow aspiration demonstrated 30-35% myeloperoxidase-positive blasts with dysplastic megakaryocytes. A bone marrow biopsy showed 70% cellularity with extensive interstitial infiltration by blast cells, increased eosinophilic precursors, and plasma cells, along with reduced mature myeloid and erythroid cells. The blast population was positive for CD38, CD117, CD33, CD68, CD7, and TdT. Based on these findings, three weeks after surgery, the patient was diagnosed with acute myeloid leukemia and subsequently underwent 16 cycles of chemotherapy followed by allogeneic bone marrow transplantation. The patient is currently under follow-up.

## Discussion

Vaginal myeloid sarcoma is an exceptionally rare entity, and its diagnosis and management require careful attention to clinical presentation, pathological features, and therapeutic strategy. As an extramedullary manifestation of myeloid proliferation, the disease often presents a diagnostic challenge due to its nonspecific and sometimes misleading clinical features, which can resemble benign conditions or more common gynecologic malignancies [8]. In our patient, the initial symptoms (including dyspareunia and the detection of a palpable vaginal mass) were subtle but characteristic, consistent with previous reports describing reproductive tract myeloid sarcomas that may precede or occur concurrently with AML [4].

Accurate diagnosis relies heavily on histopathology and immunophenotyping because clinical findings and imaging alone are rarely definitive. The tumor’s histological appearance, characterized by sheets of immature myeloid cells, can mimic lymphoma, poorly differentiated carcinoma, or even sarcoma, leading to potential misinterpretation, particularly when the site of involvement is as unusual as the vaginal wall [2,8]. In such cases, a broad immunohistochemical panel becomes indispensable. The diffuse expression of CD45, CD33, CD43, and BCL-2 in our case was instrumental in confirming the myeloid origin and ruling out other hematolymphoid or mesenchymal neoplasms.

The involvement of the vagina by myeloid sarcoma is extremely uncommon, with only a small number of cases reported in the literature [2,4,8]. Most of these occur alongside systemic disease, but in a significant subset, the extramedullary tumor may appear months before any evidence of bone marrow involvement, underscoring the need for a thorough hematologic work-up once the diagnosis is established [4]. Importantly, extramedullary presentation has been associated with a more aggressive clinical course, higher relapse rates, and poorer overall survival compared to AML confined to the bone marrow [7].

Currently, there is no standardized approach to the management of vaginal myeloid sarcoma, largely due to the rarity of reported cases and the absence of prospective studies. Surgical excision is frequently performed to obtain a definitive diagnosis or to relieve local symptoms, but it is generally considered insufficient on its own [2,8]. Most authors recommend a combined approach that includes systemic therapy similar to that used for AML in order to address potential systemic disease and improve long-term outcomes [7]. In some cases, radiotherapy may also be considered as an adjunctive option, particularly when complete resection is not feasible or when residual disease persists.

## Conclusions

Vaginal myeloid sarcoma is an exceptionally rare and aggressive malignancy that can mimic common gynecological conditions, leading to delayed diagnosis. Early identification through thorough clinical evaluation, imaging, and histopathology is critical to initiate appropriate multidisciplinary treatment. This case highlights the importance of clinician awareness and timely intervention to improve patient outcomes. Continued documentation of such rare cases is essential to refine diagnostic and therapeutic strategies. Multidisciplinary collaboration and early histopathologic confirmation of atypical vaginal masses are essential to ensure prompt diagnosis and appropriate management.
